# Evolving HIV epidemics: the urgent need to refocus on populations with risk

**DOI:** 10.1097/COH.0000000000000571

**Published:** 2019-06-24

**Authors:** Tim Brown, Wiwat Peerapatanapokin

**Affiliations:** East-West Center, Honolulu, Hawaii, USA

**Keywords:** focused intervention, HIV modeling, HIV prevention, key populations, national responses

## Abstract

**Purpose of review:**

To explore the comparative importance of HIV infections among key populations and their intimate partners as HIV epidemics evolve, and to review implications for guiding responses.

**Recent findings:**

Even as concentrated epidemics evolve, new infections among current and former key population members and their intimate partners dominate new infections. Prevalent infections in the general population grow primarily because of key population turnover and infections among their intimate partners. In generalized epidemic settings, data and analysis on key populations are often inadequate to assess the impact of key population-focused responses, so they remain limited in coverage and under resourced. Models must incorporate downstream infections in comparing impacts of alternative responses.

**Summary:**

Recognize that every epidemic is unique, moving beyond the overly simplistic concentrated/generalized epidemic paradigm that can misdirect resources. Guide HIV responses by gathering and using locally relevant data, understanding risk heterogeneity, and applying modeling at both national and sub-national levels to optimize resource allocations among different populations for greatest impact. Translate this improved understanding into clear, unequivocal advice for policymakers on where to focus for impact, breaking them free of the generalized/concentrated paradigm limiting their thinking and affecting their decisions.

## INTRODUCTION: CHANGING RESPONSES TO EVOLVING EPIDEMICS

In the late 1990s, UNAIDS and WHO introduced the concept of low-level, concentrated and generalized epidemics as a tool to guide surveillance strategies [1]. Although the concept of low-level epidemics fell by the wayside as HIV became globally ubiquitous, the paradigm of concentrated and generalized epidemics continues to dominate many people's thinking about HIV epidemics and responses to them. The original definition distinguished these two epidemic types by whether there was sustained HIV transmission within the ‘general population’, that is, that part of the population outside of certain groups perceived to be at high risk of acquiring HIV. These are groups, which are normally referred to today as ‘key populations’, and include: MSM, male and female sex workers (MSW and FSW) and their clients, people who inject drugs (PWID), transgendered individuals and prisoners and other incarcerated individuals. UNAIDS and WHO also defined simple numerical proxies for concentrated (>5% in at least one subpopulation, but <1% among urban pregnant women) and generalized epidemics (consistently over 1% in pregnant women).

As the concentrated/generalized paradigm was simple, easily defined by prevalence and heavily promoted by international technical partners, it was quickly adopted on a large scale. By the mid-2000s, it was also being used to define national responses. Countries with concentrated epidemics were told to focus on locally relevant at-risk populations; countries with generalized epidemics were to put most of the emphasis on the general population [2]. In the early 2000s, the challenge in taking this advice in concentrated settings was that UNAIDS and other partners were stressing the need for prevention and treatment to reach everyone and promoting an expanded response of prevention, treatment and mitigation efforts covering the entire population [3,4]. This was an essential part of building the successful global coalition to expand and mobilize funding for the global response in the decade after the 2000 Durban AIDS Conference, but it often resulted in failures to prioritize programs, especially programs for key populations [5]. Given the challenges in mobilizing resources for socially and politically stigmatized populations in this environment, advocates frequently seized upon a ‘concentrated epidemics will become generalized’ argument to build support for key population efforts.

Today, this underlying misconception of ‘concentrated epidemics will become generalized’ leads the press, some advocates and many decision makers controlling national resources to frame concentrated epidemics in terms of transitioning ‘heterosexual’ or ‘general population’ epidemics and to make program choices accordingly. For example: ‘In Eastern Europe, heterosexual transmission now accounts for 55% of new infections. So the epidemic is moving from key populations to the general population’ [6]. Even when followed by a call for key population programs, such statements reinforce a prevailing paradigm that can lead to bad program choices, for example, ‘Philippine rights activists for lesbian, gay, bisexual and transgender (LGBT) people blame these policy failures on the government's focus on HIV prevention policies that target heterosexual couples rather than members of the LGBT community’ [7]. This interpretation of epidemic dynamics is further buttressed by the observation that countries with long running concentrated epidemics have an increasing number of HIV cases detected or presenting for treatment from the general population. Coupled with widespread stigma and discrimination, this belief in a transitioning epidemic encourages redirection of resources and makes it challenging to obtain national/local resources for robust responses among key populations.

Countries with generalized epidemics, on the other hand, were encouraged to focus most of their resources on the general population. This led to programs in sub-Saharan Africa that focused almost entirely on the population at large with some limited attention to heterosexual transmission through sex work [8]. Although the 2007 UNAIDS guidelines on intensifying prevention in generalized settings explicitly state that ‘programmes for most-at-risk populations remain important’, they focus primarily on general population efforts. Consequently, given people's challenges in absorbing complex messages, resources flowed to general population efforts whereas key population programs in generalized epidemics remained limited in scope, funding and coverage. This persists today [9–12]. The corollary to ‘concentrated becomes generalized’ is ‘once epidemics become generalized, key populations aren’t too important’. This has become a self-fulfilling prophecy as the lack of interest in key populations in generalized settings has restricted the collection of data on prevalence, size and risk behaviors until recently, making it difficult to accurately assess the proportion of new infections occurring among key populations and their immediate partners [13^▪▪^]. 

**Box 1:**
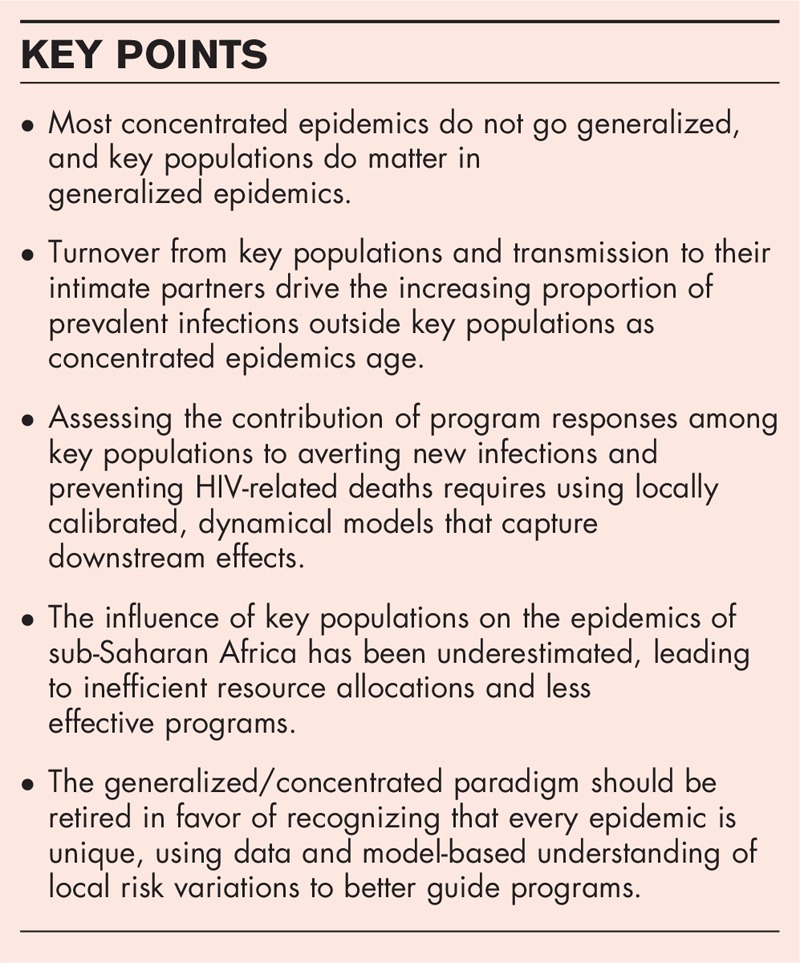
no caption available

## Goal of this review

The goal of this review is to improve understanding of how to focus responses in different epidemic settings for maximum effectiveness. It, thus, addresses three directly relevant questions arising from the discussion above:

(1) Given increasing general population case reports in long-running concentrated epidemics, should the response emphasis shift from key populations to the general population?
(2) How can the influence of key populations in generalized settings be characterized and are current responses adequate?
(3) What do data, models and analyses say about these two questions and how can they be applied to better inform key decision makers of the role of key populations in local epidemics?

## THE EVOLUTION OF CONCENTRATED EPIDEMICS: A REGIONAL CASE STUDY

Why does the proportion of reported infections from the general population increase in long-running concentrated epidemics and what are its implications for prevention efforts? Models are best positioned to answer this question. The AIDS Epidemic Model (AEM) is a concentrated epidemic model originally developed for Asian countries [14]. AEM contains most key populations: male and female sex workers and their clients, MSM, people who inject drugs and transgendered populations. It also includes nonkey population men and women. AEM models the various routes of transmission among and between these populations, including heterosexual transmission through noncommercial casual sex and sex between intimate partners. AEM is widely used throughout Asia; 11 countries used it to prepare the national models submitted to UNAIDS in 2018. AEM tracks current and new infections, sources of infection, and turnover for each population in the model, providing a regional ‘laboratory’ for exploring the natural history of these epidemics.

### The evolving epidemics of Asia

**FIGURE 1:**
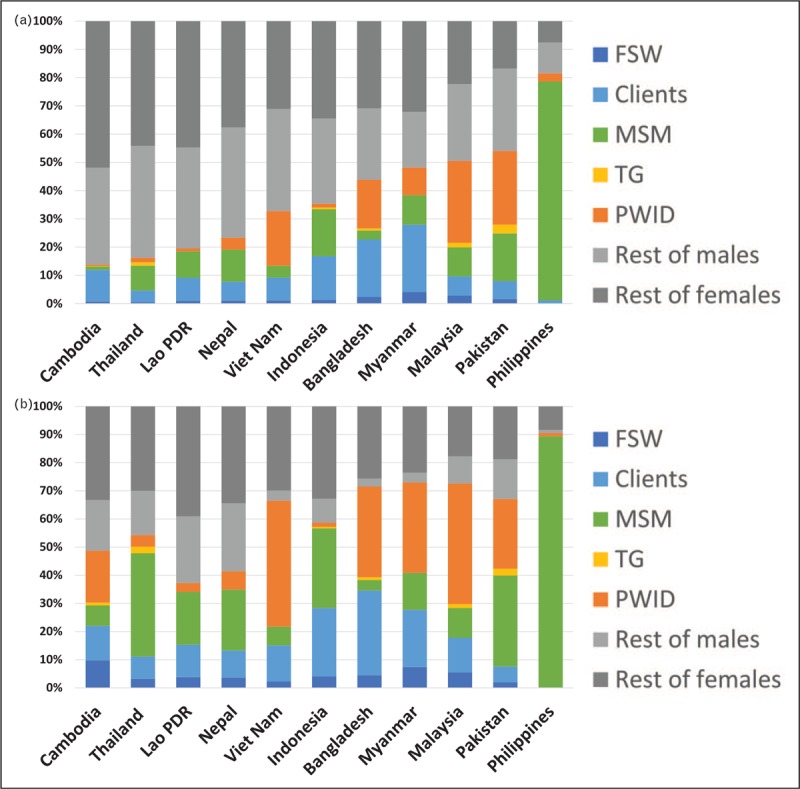
(a) The number of prevalent, that is, current, HIV infections in 11 Asian countries in 2017 by subpopulation, and (b) the number of new HIV infections by subpopulation. The countries have been ordered from left to right based on the proportion of current infections among key populations. The figures are derived from national models developed by local AEM teams as submitted to UNAIDS in 2018. Values are for female sex workers (FSW), clients, MSM, transgenders (TG), people who inject drugs (PWID) and the rest of the male and female population. AEM, AIDS Epidemic Model.

Figure 1 shows the number of (a) current infections and (b) new infections in the 11 countries in 2017 by population. The proportion of current infections outside key populations varies from 18 to 86%, whereas the proportion of new infections arising outside key populations varies from 10 to 62%. The first thing of note is the incredible diversity in patterns of new infections by key population between the countries. In five countries, people who inject drugs account for one-third or more of new infections. In the Philippines, the epidemic is almost completely among MSM, whereas in other countries, the proportion of new infections among MSM is smaller but growing. New infections among sex workers and clients now account for only 19% of new infections in these countries, although it varies from 0.1 to 35% across the countries shown. Prevention resources must be allocated differently in these countries to maximize their impact and multiple AEM scenarios for different program mixes are typically prepared to inform decision makers of the most impactful combinations. The second important observation is that countries with the longest running epidemics, that is, Thailand and Cambodia, have the largest proportion of current infections among the general population, shown on the graphs as ‘Rest of males/females’. They also show a large portion of new infections, roughly 50%, among the general population. In the more recent and growing epidemics, for example those in Pakistan and the Philippines, most new infections occur among key populations and a smaller proportion of current infections are found among the general population.

**FIGURE 2:**
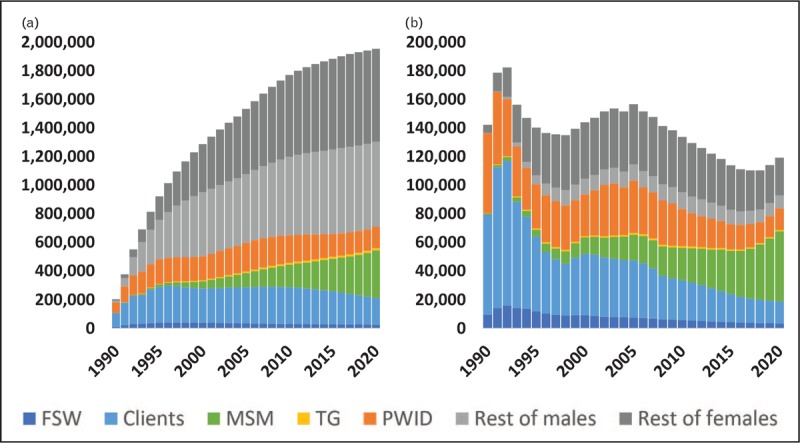
The evolution of (a) current infections and (b) new infections by subpopulation from 1990 to 2020 aggregated across the 11 AIDS Epidemic Model countries. The gray bars show the evolution of current and new infections in the general population (rest of males/rest of females).

Figure 2 shows the combined growth of current and new infections for these 11 countries. At a regional level, the proportion of current infections among the general population grows steadily as epidemics run longer; by 2018, 65% of current infections are outside key populations. However, the proportion of new infections outside key populations never rises above 35%. The other important observation about HIV in the general population is that while current infections are almost equally divided between men and women, new infections among women outnumber new infections among men by a factor of 3 to 1. This raises several important questions:

(1) With two-thirds of new HIV infections occurring among key populations, how can two-thirds of current infections be found outside them?
(2) Why does the proportion of current infections outside key populations increase with epidemic age? Is transmission in these epidemics ‘going generalized’ as many believe?
(3) Why is the number of new infections among women outside key populations three times that for men?

### How key populations influence epidemic dynamics

Answering these questions requires a deeper insight into the varied roles key populations play in the dynamics of concentrated epidemics. Key populations influence the evolution of these epidemics in four ways:

First, if no protective steps are taken, HIV transmission among key population members is highly efficient. Transmission risk in key populations is elevated for several reasons: the high-efficiency of anal sex in transmitting HIV for MSM and transgendered populations [15]; high-frequency of needle sharing acts among many PWID; substantial partner exchange rates and the facilitating role of other sexually transmitted infections and primary HIV infection for sex workers and clients. Vaginal sex, especially with much lower rates of facilitating sexually transmitted infections outside certain key populations, transmits HIV at significantly lower rates and over a much longer time span [16]. These factors give key populations a disproportionate role in epidemic dynamics, thus their larger contribution to new infections.

**FIGURE 3:**
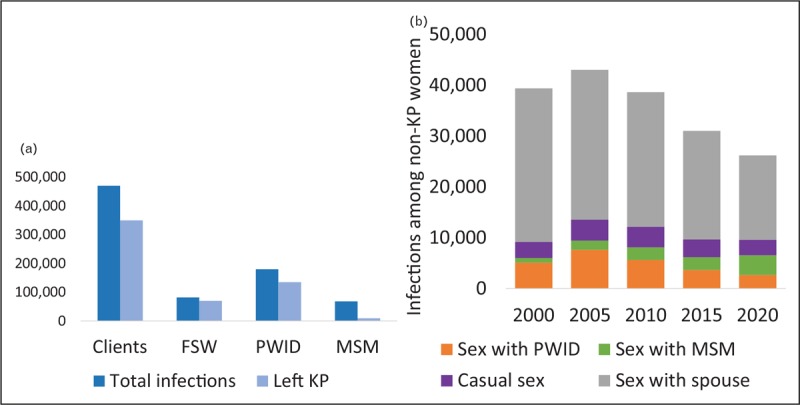
(a) The number of HIV+ individuals leaving each key population to return to the ‘general population’ in Thailand from the start of the epidemic to 2017, and (b) infections among nonkey population women in the 11 countries over time by route of infection.

Second, key populations are not closed; people move in and out of them [17]. A young woman may become a sex worker for several years, but then returns home to marry and open a shop. Young men may be particularly active as clients, but then stop visiting sex workers after marriage. Many PWID stop injecting after several years. At this point, these former members of key populations return to the ‘general population’. Infections that occurred while they were at elevated risk may be detected long after they have re-entered that general population. AEM tracks this turnover between populations. Figure 3a shows the proportion of HIV infections occurring within key populations that have since returned to the general population for Thailand, one of the longest-running epidemics in Asia. Through 2017 almost 502 000 HIV-positive men who contracted HIV while in key populations have returned to the general population, whereas only 52 000 new infections have occurred directly within the male general population. Cumulatively, 91% of the infections found among the male general population originated from transmission within key populations. For women, the effect is less pronounced as cumulatively only 71 000 HIV-positive sex workers returned to the female general population compared with 311 000 infections occurring among these women, that is, 19%. This movement of HIV-positive individuals from key populations partially explains the growing proportion of general population prevalent infections as epidemic ages. In terms of newly detected infections in each population, the effect appears even larger as many older infections are only diagnosed after progression to symptomatic illness, that is, long after the person leaves a key population.

Third, members of key populations often have intimate partners to whom HIV may be transmitted, both while in key populations and after transitioning out of them. For the 11 AEM countries, Fig. 3b shows the largest proportion of new infections among women not in key populations comes from sex with their current spouse; however, as described in the preceding paragraph, most infections among these men were acquired when they were members of key populations. This is the other piece of the increase in prevalent infections in the general population: vastly more ex-members of key populations are men and pass HIV onto their spouses, contributing to an increase in prevalent infections among general population women.

**FIGURE 4:**
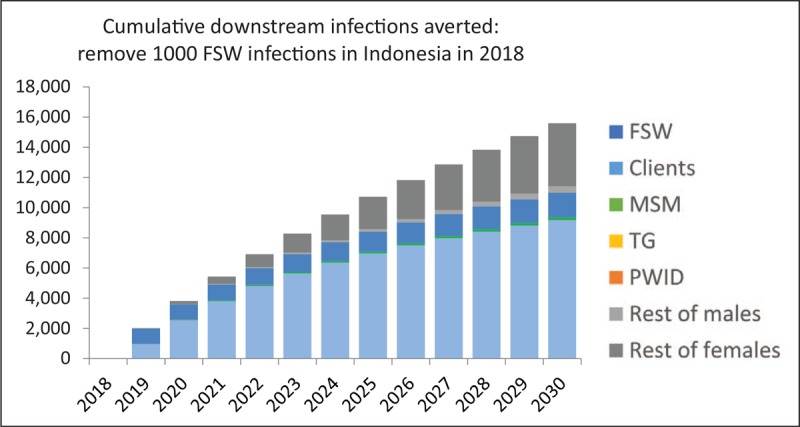
Downstream infections averted in the Indonesia model if 1000 female sex worker infections are prevented in 2018. By 2030, this will avert 9200 infections among clients, 640 additional infections among sex workers, 420 infections among males not in key populations and 4200 infections among females not in key populations.

Fourth, the infection of wives and other intimate partners is an example of how infections occurring among key populations can result in substantial numbers of downstream transmissions. Figure 4 shows the impact of averting 1000 infections among female sex workers in 2018 for Indonesia. For the first few years, most of the averted infections are among clients. However, as clients and ex-clients then more gradually transmit HIV to their current and future wives, the number of infections averted among women not in key populations increases over time, a pattern first observed by Weniger *et al*. [18] in 1991 in Thailand that has repeated throughout Asia. When there is a substantial nexus between injecting drug use and sex work, an epidemic among PWID can jump start the sex work component of an epidemic, producing large numbers of downstream infections [19,20].

These dynamic factors provide answers to the questions raised earlier. Most of the new infections occur in key populations because of the more efficient transmission among them, but most of those infections eventually become prevalent general population infections through turnover. In addition, HIV is transmitted to their predominantly female intimate partners who are not themselves key population members. In the countries modeled here only 2.5% of cumulative HIV infections through 2017 occurred through casual sex, that is, sex outside of relationships that were not sex work-related. The proportion of current infections outside key populations increases over time because turnover accumulates faster than mortality effects. Increasing rates of ART coverage, which greatly extend people's lives, increase this effect. Thus, the longer an epidemic runs the greater the proportion of current infections that are outside key populations. New infections among women outside key populations greatly outnumber those of men because most arise from male partners who are or were members of key populations and the number of men in key populations vastly exceeds the number of women. In the 11 countries described here, there were 24 million clients, 740 000 PWID, 4 million MSM, but only 1 million FSW.

This picture has important implications for prevention efforts in today's concentrated settings. First, because most infections in the general population originate from infections within key populations or transmission to their intimate partners, a shift to general population prevention programs would be a major mistake. Instead, the focus must be kept on key population prevention; this will have the greatest downstream impact on total infections, including those in the general population, especially women. Second, programs for intimate partners of key populations should be an essential component of all prevention efforts. It would also be valuable to develop innovative programs focused on encouraging former key population members to test and, if positive, begin antiretroviral treatment and be supported in offering testing to partners through assisted partner notification services. These efforts would address most infections occurring outside key populations. Finally, in allocating resources for prevention programs, look not only at the current distributions of new infections, but also consider downstream infections in identifying programs with the greatest impact.

## CONCENTRATED EPIDEMICS IN OTHER REGIONS: SIMILAR BUT DIFFERENT

Globally, the ‘concentrated’ regions are Asia and the Pacific, the Middle East and North Africa (MENA), Eastern Europe and Central Asia (EECA), and Western and Central Europe and North America (WCENA). Latin America and the Caribbean (LAC) are a mix with Latin America being predominantly concentrated epidemics, whereas the Caribbean has more generalized epidemics. Does the situation described for Asia apply in other concentrated epidemic settings, that is, are the dynamics likely to be the same in the other regions considered concentrated?

**FIGURE 5:**
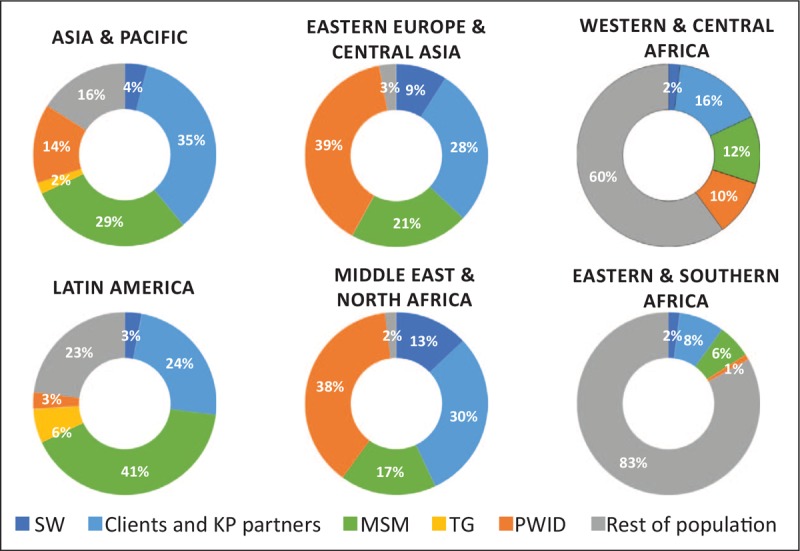
Distribution of new infections by sub-population in different regions of the world in 2017 as estimated by UNAIDS. Source: Miles to Go 2018 [21].

UNAIDS analyzed the data from national models to estimate the proportion of new infections in 2017 occurring among key populations and their immediate partners in each region, the result is shown in Fig. 5[21]. The combined proportions for sex workers, clients and key population partners are quite similar for Asia and the Pacific, EECA and MENA (37–43%). Latin America has a smaller proportion from these groups, but almost twice the proportion from MSM, whereas the epidemics in EECA and MENA are much more strongly influenced by PWID than the other regions. Latin America also shows a significant proportion among transgendered populations. In WCENA (not shown), the proportion among MSM is even larger, 57%.

Work with dynamical models in these regions has tended to focus on the most important key populations in the region or country as revealed by surveillance data. The work in Latin America has primarily explored programs for MSM [22–27] and transgender women [28], although some modeling work has looked at FSW and PWID [29–31]. In EECA, the modeling is strongly skewed toward PWID populations, given the dominant role of injecting in new infections [32–35]. And in MENA, modeling work has focused on injecting [36] with some modes of transmission work in Morocco, which did highlight significant new infections from sex work [37,38].

Most of these efforts did not use models incorporating all populations but focused on analysis of effectiveness and cost-effectiveness of programs within specific populations, such as MSM or PWID. However, there has been substantial work done with Optima in EECA and Latin America [39^▪▪^]. Optima is a full dynamical model, incorporating the effects of downstream impacts in identifying the most effective programs [40]. In normal country applications, both key populations and the general population are included. If HIV spread outside key populations were becoming a significant contributor, one would expect these analyses to recommend increases in prevention efforts, for example, condom promotion among the general population. This was not seen – in fact, the recommendations in 11 countries in EECA and 4 countries in Latin America were to scale up or sustain one or more key population programs while scaling down general population and youth programs [39^▪▪^]. Given these results and the general similarity of the regional patterns of new infections in epidemics that have been ongoing for two to three decades, it seems unlikely that transmission outside key populations and their intimate partners plays a major role.

## THE CHALLENGES OF ESTIMATING KEY POPULATION EFFECTS IN GENERALIZED SETTINGS

Evaluating the role of key populations in the generalized epidemic settings of sub-Saharan Africa (SSA) is more challenging. In some cases, the key populations themselves are more poorly defined and less visible than in concentrated settings. For example, ‘sex work’ often involves a mix of self-identified, more active, professional sex workers in diverse settings [41] and less easily identifiable, more occasional, women engaging in transactional sex [42,43]. MSM in SSA face severe stigma and discrimination, keeping them out of sight and limiting programs, and the situation is worsening with the regional resurgence of conservative attitudes [44,45]. Although there is growing awareness, with 36 out of 47 countries reporting some evidence of injecting drug use in 2017 [46], only 7 countries had any needle and syringe programs for people who inject drugs [9].

### Comparative prevalence among key populations is high whereas size estimates are often low

**FIGURE 6:**
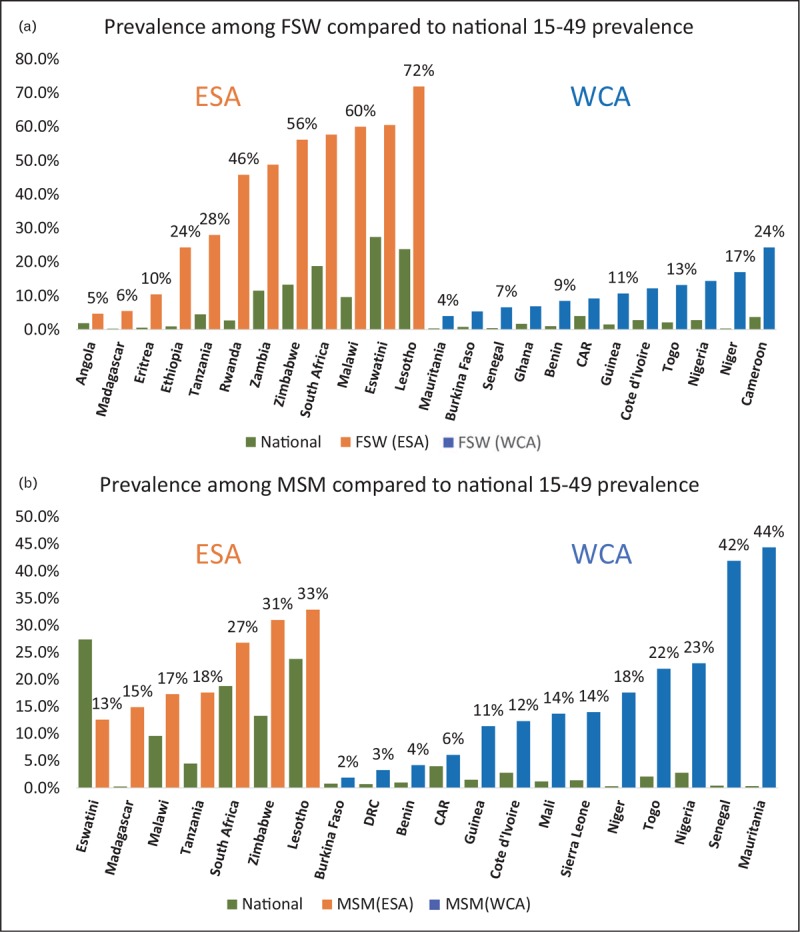
HIV prevalence among (a) Female sex worker and (b) MSM compared against national prevalence among those aged 15–49 years for Eastern and Southern Africa (ESA in orange) and Western and Central Africa (WCA in blue). Although not nationally representative in most cases, they give an idea of the range of values being observed in the regions. Source: UNAIDS Data 2018 [47].

In recent years, key population data collection has increased, revealing that members of key populations in the region are at elevated risk for HIV. Figure 6 compares HIV prevalence for FSW and MSM as reported to UNAIDS with national prevalence [47]. The Eastern and Southern Africa (ESA) region reports some of the highest prevalence among sex workers in the world, whereas reports in Western and Central Africa (WCA) are lower, but still substantial. The median ratio of FSW to national prevalence in ESA and WCA is 5 and 7, respectively. Prevalence among MSM is high in both regions, but with a higher median ratio for MSM to national prevalence of 8 in WCA versus 2 in ESA. Among PWID, prevalence in the 4 ESA countries reporting data varies from 8.5 to 46.4%. Although 10 WCA countries report generally lower prevalence among PWID, varying from 1.6 to 10.2% [47].

**FIGURE 7:**
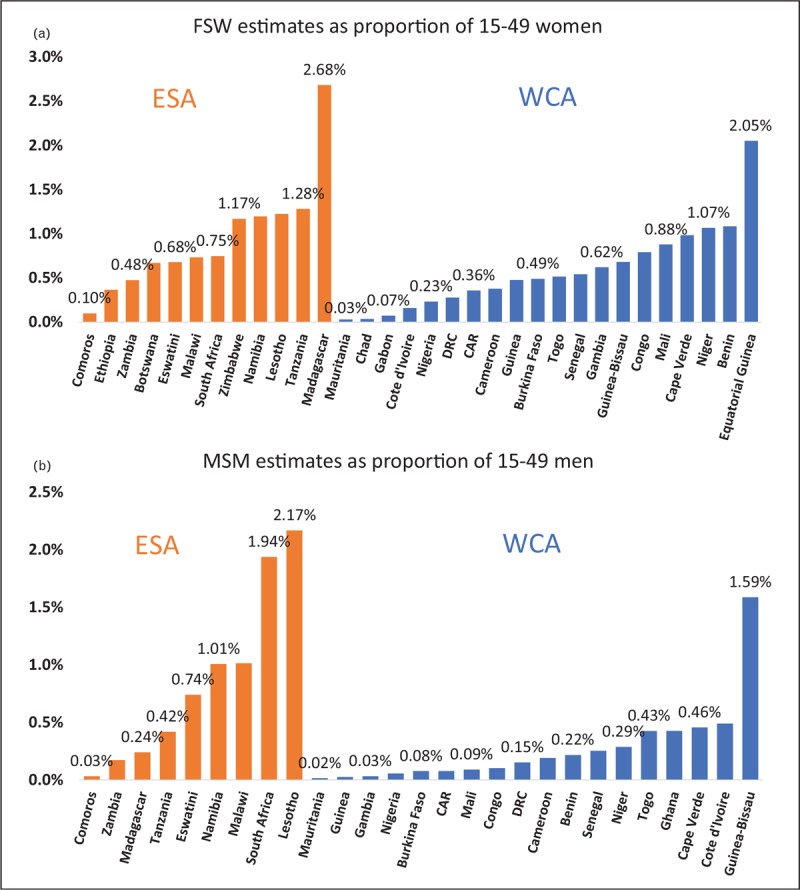
Size estimates for (a) Sex worker and (b) MSM as a proportion of 15–49-year-old population of the same sex for Eastern and Southern Africa (ESA in orange) and Western and Central Africa (WCA in blue). Source: UNAIDS Data 2018 [47].

Another critical input in assessing the contribution of key populations in SSA is their size, shown as a proportion of the population in Fig. 7. Standard techniques for estimating key population size, for example, census/mapping, multiplier or capture–recapture [48], present their own challenges in sub-Saharan Africa. Resource constraints on key population programs often limit the ability to do frequent or large-scale mapping. Multiplier methods can underestimate numbers if key population program data are limited or populations are unrecognized and/or inaccessible to surveyors, for example, when stigmatized population members misreport their risk. These issues have been observed in size estimation estimates in the region for both MSM and sex workers [49,50]. Serious efforts to improve size estimates have often used multiple methods [51–53] or applied innovative approaches, for example, Bayesian techniques applied to RDS multipliers [54] or combining venue-based sampling and RDS [55].

The increasing global emphasis on quality program data [56] presents another potential source for size estimates, but it requires de-duplication across multiple service providers and venues. However, this faces major hurdles: obtaining accurate information from program clients on their key population status, storing this information so as to ensure confidentiality and prevent abuse, and developing unique identifiers for service improvement and de-duplication [57]. High levels of discrimination and social stigma almost inevitably lead to under-reporting and low size estimates [58]. Although biometrics have great potential as unique identifiers, they have sometimes been rejected by affected communities because they fear abuse by authorities [59].

Despite these challenges, the number of size estimates is growing. On average, ESA countries providing estimates report larger proportions of the 15–49 population being FSW or MSM than WCA countries. The estimates for FSW are mostly in agreement with past reports of regional size ranges [60] and data from more recent reviews [61]. However, in both regions, estimates for MSM tend to be quite low, mostly less than 0.5% of 15–49 men, and in several cases less than 0.1%. These are much lower than are biologically plausible [62] or are seen in more systematic data collection in other regions [63]. Social media-based estimates of same-sex interest in Africa also give much higher estimates than reported to UNAIDS [64^▪^]. As Davis *et al.*[58] have highlighted, countries criminalizing same-sex behavior often report lower proportions of MSM in the adult population, and many of the countries of sub-Saharan Africa criminalize and/or exhibit extensive homophobia [65]. Estimates of people who inject drugs for the region vary from 0.14 to 1.0% of males aged 15–64 years, with 11.6% of PWID reported to be women [46].

### Early attempts underestimated the influence of key populations in generalized settings

The earliest widespread estimates of the impact of key populations in SSA occurred in the mid-2000s using the Modes of Transmission (MOT) model, which estimates the proportion of annual new infections acquired by sub-populations based on prevalence, size and some limited behavioral data [66]. MOT studies in ESA showed between 60 and 95% of new infections occurring among the ‘general heterosexual population’ with another 7–15% attributed to sex work. Contributions from PWID and MSM were at most a few percent. In Western Africa, a higher contribution of new infections because of sex work was observed, 10–32%, but still 54–72% of new infections were among the general population. Contributions from key populations were larger than in ESA, with between 1 and 12% among MSM and 1–8% among PWID. These estimates depend critically on both prevalence and size estimates, meaning the limited availability of representative prevalence data and the skew toward lower size estimates for some populations may misrepresent the magnitude of contributions. It is worth noting that the MOT results showed substantial inter-country variation in the contributions of different groups to new infections, just as AEM did in Asia.

From the late 2000s through the mid-2010s, thinking about the epidemics in West and Central Africa evolved. Although ‘generalized’ by the 1% in pregnant women definition with MOT analyses showing the most new infections among general population categories [67,68], closer examination of local data in many countries of the region called into question whether the heterosexual components of the epidemic were self-sustaining in the absence of key populations [69]. Circumcision was more common in WCA than in ESA, which tends to reduce transmission, and national prevalence was generally lower. It was also recognized that the traditional generalized/concentrated prevalence distinction failed to align programming with epidemiology in the countries of WCA, which called for more, but not exclusive, emphasis on key populations [70]. This led some to redefine concentrated epidemics as ones where transmission would not be sustained in the absence of key populations, such as sex workers, clients, MSM and PWID [71] and to the increasing use of the term ‘mixed’ epidemics by others, where both key population and general population transmission were active [72]. In either case, many began thinking of the epidemics in some WCA countries as concentrated and/or mixed, but others without in-depth understanding of the epidemiology and what the data were telling them continued to think of them as generalized.

In the mid-2010s, the MOT model was correctly criticized as a ‘static’ model, giving only a cross-sectional snapshot of incidence and failing to capture the true population level impact of interventions to reduce new infections among key populations [73–75]. Another critique was of its focus on those acquiring HIV rather than those transmitting it, which were the logical place to direct interventions. Thus, it had limitations in allocating prevention and treatment resources among different sub-populations to maximally reduce future infections and deaths. The core of this criticism was that the MOT model did not include the downstream benefits in future averted infections that accrue from preventing an infection in a key population today, as discussed earlier.

### Dynamic models of the influence of key populations change the picture

Mishra *et al.*[76] reviewed the importance of incorporating downstream infections in assessing the impacts of sex work interventions in generalized epidemic settings. They found median estimates of the contribution of sex work to the epidemic, combining infections among sex workers and clients from static, MOT-style exercises (9%) were much lower than those coming from a limited set of dynamical models available at the time (14–38% with sex work interventions and 58–89% without) [77–79]. Accordingly, they recommended summing new infections in sex workers and clients with the resulting downstream infections from chains of transmission to others over many years to more fully capture the longer term benefits of preventing sex work infections today. This is in fact what is routinely done now in cost-effectiveness and resource allocation exercises in sub-Saharan Africa. Dynamical models, such as Goals, Optima and EMOD are used to estimate the total number of infections and/or deaths in all populations over some extended time frame, for example, 10, 20 or 30 years, under alternative resource allocation scenarios, including those addressing key populations [39^▪▪^,^80^^▪▪^]. Comparing such impact estimates inherently incorporates downstream infections into identifying the most efficient and effective intervention mix. However, doing this comes at the cost of meeting the more complex input data requirements of dynamical models, including prevalence, behaviors and size estimates, and calibrating the model for trends over time in the various populations.

Retrospective modeling including downstream infections makes the important early contributions of sex work to generalized epidemics apparent. In modeling Kisumu, Kenya from 2000 to 2020 with STDSIM, Steen *et al.*[78] found that removing sex work entirely would have reduced incidence by 66% and prevalence by 56%, but still resulted in a self-sustaining heterosexual epidemic. Sex workers were divided into high, medium and low contacts, with the high group being more visible sex workers and the low contact group representing women engaging in transactional sex. Of interest, they found that intervention among the high group alone accrued almost the same benefits as reaching all sex workers. A similar analysis in Côte d’Ivoire showed that over time, the 10-year population attributable fraction (PAF) of infections because of sex work dropped from 95% in 1976–1985 to about 19% in 2005–2015, decreasing as the epidemic became more established in the heterosexual population [81^▪^]. Another model for Bobo-Dioulasso, Burkina Faso found the 5-year PAF for sex work dropping from 75 to 88% from 1985 to 1990 to 39% from 1995 to 2010 as condom use rose among sex workers [77]. The study also found a higher PAF of 60–70% associated with full-time sex workers compared with 10–20% for occasional sex workers, reinforcing the findings of Steen *et al.*

Other than Goals-based and Optima-based models in the region, which routinely include all key populations whenever data are available, other dynamical models including MSM and PWID are rarer than those that focus on sex work and much more geographically limited. However, as incidence in heterosexual populations is reduced through ART and prevention efforts, these models do show that addressing the needs of key populations will likely become increasingly important to effective resource allocation in some places. Mukandavire *et al.*[82^▪^] prepared models for Dakar, Senegal, a city with 6% prevalence in FSW and 30% in MSM in 2016. They found same-sex behaviors to be the primary contributor to new infections in 2015, 51.4% compared with 13.8% through sex work. Meeting MSM's prevention needs would avert 64.1% of new infections over the next 10 years, while strengthening sex work programs would only avert 13.6% because of existing long-term prevention efforts for and high ART coverage among FSW. Cremin *et al.*[83] modeled Nairobi, Kenya where the overall epidemic is in decline, but an epidemic persists among MSM and male sex workers (MSW). Their in-depth model, based on extensive data collation in Nairobi and fitting to historical data in each group, included various heterosexual groups, multiple risk categories for sex workers, and MSM, but excluded PWID. Given continuing declines in incidence among heterosexuals and sex workers, their analysis identified the optimal portfolio for prevention as one focused first on condom promotion for MSM and MSW followed by strengthening ART efforts and then other interventions. Maheu-Giroux *et al.*[81^▪^] did a similar exercise in Côte d’Ivoire constructing a detailed historical model, also excluding PWID, that fit observed trends. They found that achieving 90–90–90 targets among sex workers, clients and MSM alone could achieve a 30% reduction in new infections, compared with 50% if the targets are reached for all [84]. This key population-focused approach was the most cost-effective of multiple scenarios examined [85^▪▪^]. Recently phylodynamic approaches are being combined with modeling to look at the downstream impact of key populations. Volz *et al.*[86^▪▪^] explored the influence of MSM on the epidemic in Abuja, Nigeria, finding that 9% of infections of women were from partners who were MSM in 2014. Because of downstream effects, they found that focused treatment for MSM could avert 27% of infections over 20 years, compared with 54% for universal test and treat, making a targeted approach much more cost-effective.

Outside of Goals and Optima work, little has been done on dynamical models for people who inject drugs, mirroring the limited PWID programs noted by Larney *et al.*[9]. Rhodes *et al.*[87] built a model to look at the effects of methadone programs in Kenya, but it had a limited sexual transmission model that did not situate PWID in terms of overall influence on the epidemic. Monteiro *et al.*[88] did include the influence of people who used drugs in Cabo Verde, but stressed the need to strengthen surveillance among people using drugs to detect if injection practices start and then add injecting drug use into the model.

### Geographic risk heterogeneity is important to planning effective responses

Although the preceding sections imply the contribution of key populations in WCA is generally greater than in ESA, such generalizations are not enough to direct programs appropriately. The influence of key populations varies greatly between and within countries. McGillen *et al.*[89] ran dynamical models for 18 high-burden SSA countries broken into 203 sub-national regions and found substantial heterogeneity in risk by different sub-populations and by geography. Their analysis showed focusing prevention on marginalized populations in those countries could avert 70% more infections than a strategy based on current less-focused targeting approaches, but the most effective combination of program components varied from one sub-national region to the next. In a modeling analysis of six counties in the Nyanza region of Kenya, Bershteyn *et al.*[90] found substantial geographic heterogeneity in the contribution of sex work to transmission, requiring different intervention mixes to efficiently curtail transmission. Similarly, a Goals model analysis of the NSP in Mozambique found that in the North sex work interventions would have the greatest impact, whereas in the Center and South, voluntary male medical circumcision would be most impactful [91]. Although there has recently been a substantial focus on geospatial variability in prevalence and incidence in targeting responses [92], these studies highlight that it is also important to focus on sub-national risk heterogeneity, including risk among key populations, to optimize prevention investments in each location. However, the knowledge base at present is insufficient to most efficiently allocate prevention resources [10]. This increases the importance of recent efforts to systematize this data and make it more readily available for program planning, analysis and modeling [13^▪▪^].

### Data for key populations remains limited

The more in-depth analytic models in Senegal, Kenya and Côte d’Ivoire were each based on extensive data collection exercises, gathering both current and historical data on prevalence, behaviors, programs and size estimates in both key populations and those outside them [81^▪^,82^▪^,^83^]. However, extensive reviews of the data in West and Central Africa have found that whereas some data and program targets for sex workers were often available, much less data was available for MSM and that data for PWID was rare and incomplete [10,93]. The situation has been slowly improving over time, and the improvements in data have increased UNAIDS static estimates of the proportion of new infections among key populations and their immediate partners in WCA from 27% in 2014 to 40% in 2017 [21,94]. Degenhardt *et al.*[46] document the growth of PWID data in the countries of SSA between 2007 and 2017, but few countries have translated this into programs [9].

Yotebieng *et al.*[95] and Wolf *et al.* stress how the lack of key population data along the treatment cascade (prevalence, size estimates, facilitators and barriers) at national and sub-national levels in SSA, presents a major barrier to the implementation of effective ‘treat all’ strategies for these groups [96]. It also contributes to an absence of modeling work addressing ‘treat all’ for key populations, highlighted by Kimmel *et al.*[97]. Past reviews have stressed the serious deficit in cascade information for key population, both in SSA and globally [98,99], and efforts are underway to improve this knowledge base [96]. The cascade is the next major focus for modelers as identifying gaps along the cascade and estimating the effects that programs can have will impact both transmission among and quality of care for key populations and their partners.

The need for this data to guide programs, inform models to target them appropriately and scale-up programs to meet key population needs is urgent. This makes the effort by the Global HIV Research Group to systematize the collection of key population data globally and disseminate them in easy-to-use forms a critical first step in identifying ongoing gaps and translating key population data and models into actions and accountability [13^▪▪^]. As this data comes online, modelers should use it to expand the number of countries in SSA with dynamical models that include key populations and improve models in countries already doing so. The gaps identified must guide data collection efforts to ensure that key populations are appropriately reflected in future national models and strategic planning.

## CONCLUSIONS AND IMPLICATIONS FOR IMPROVING HIV RESPONSES

The analysis here shows that for most ‘concentrated’ epidemics outside of sub-Saharan Africa, key populations must remain at the core of epidemic responses. Although the proportion of prevalent HIV infections among the ‘general population’ grows over time, this growth is primarily reflecting turnover in key populations and transmission from current and former key population members to their intimate partners. It is not that HIV is being heavily transmitted within the general population other than to intimate partners of key populations, but that HIV acquired while in key populations is currently found in the ‘general population’. Only a limited amount of transmission is between members of the general population with no relationship to key populations. So, in most cases, concentrated epidemics do not go ‘generalized’.

This has major implications for responses in what we consider to be concentrated epidemics today:

(1) The focus in prevention efforts should remain on key populations and not shift to general population efforts targeting lower risk populations. Resources spent on general population efforts carry major opportunity costs in forgone averted infections and deaths among the key populations and their intimate partners.
(2) Programs for key populations should expand efforts and research to prevent transmission to the intimate partners of current and former key population members, including clients. Research should be undertaken to develop innovative programs to reach HIV-positive former key population members for testing, ART access and partner referrals for testing and PrEP.
(3) Program resources should be allocated among populations in a way that maximally reduces downstream infections and deaths considering each country's unique epidemic situation.

In the epidemics of sub-Saharan Africa, which most still view as generalized, the expanding database clearly demonstrates that key populations have greatly elevated prevalence compared with the population at large. Current responses are not adequate or equitable and key populations continue to disproportionately feel the impacts of HIV. Moreover, static analyses, such as the Modes of Transmission model underestimate the influence of key populations on epidemics by not accounting for downstream infections, making mobilization of the necessary responses difficult. In the case of sex work, modeling has shown the magnitude of downstream contributions can be large, especially in early stages of generalized epidemics. Even today, expanding program coverage for sex workers, proven effective in reducing transmission, will pay substantial benefits at comparatively low cost in most countries. Yet, coverage in many countries remains far from universal [11].

Too little attention has been paid to the needs of MSM and PWID in sub-Saharan Africa, two populations with even higher prevalence compared with the population at large than sex workers. This has been aided and abetted by a lack of reliable data on prevalence, size estimates and risk behaviors and serious stigma and discrimination. Although data are improving in recent years and efforts to systematize and make this data accessible are underway [13^▪▪^], the next major challenge will be using this data to construct models accurately reflecting the overall impact these groups have on local epidemics and then disseminating this information widely for decision-making. As ART and prevention efforts bring down incidence outside key populations, these populations will likely play an increasing role in new and downstream infections and deaths, especially as they often have less access to treatment and prevention. Once again, the truism is wrong, key populations do matter in generalized epidemics.

It is time to put the concept of generalized and concentrated epidemics to rest; it has outlived its usefulness. It encourages those not grounded in HIV epidemiology to assume a static view of dynamic epidemics, when the drivers of the epidemic are evolving over time as behaviors, the effects of prevention and expanded treatment programs, and prevalence among different populations change. This in turn leads to simplistic and inefficient choices about where to focus available resources, based on an incorrect picture of the epidemic. It is also time to bury the concept of ‘regional’ and ‘national’ epidemics. In each of the regions, while there may be aggregate patterns, when one looks at the country level, the contributions of key and nonkey populations varies greatly from country to country, affected by the patterns of risk and the impacts of past prevention and treatment programs. In every region, the epidemics are as diverse as the histories and cultures of the countries and they require different responses. Even within a country, epidemics are rarely homogeneous; the intensity and drivers of the epidemic vary from place to place and that calls for varying responses. Expanded work to identify sub-national risk patterns and assess local key drivers is needed to adjust the response in different areas to maximize effectiveness. This may require developing simplified models or easy to apply algorithms that are more accessible to local health authorities.

Instead, these simplistic conceptions of epidemics should be replaced by the recognition that ‘each epidemic is unique’. This should be engendered in a systematic approach focused on understanding local variations in risk and prevalence between and within countries and between and within sub-populations. Starting by identifying and filling important data gaps for all local populations, countries should then apply dynamical models to appropriately target and adapt responses as epidemics evolve. These models must include all important populations, including key populations, even in countries where people think them unimportant. One of the lessons learned in the Global Estimates process is that building something into models drives the data generation process, and until the data weaknesses for key populations are addressed, either by improving data systems or using expanded program data, their role in epidemics and their programmatic needs will not be properly assessed and met. Accordingly, all relevant key populations should be included in future modeling work regardless of region or country, even if informed assumptions need to be made in the short-term. With more comprehensive data and models, resource allocations can be equitably adapted for maximum impact as epidemics evolve over time.

Finally, improving data and building detailed models explaining local epidemiological realities does no good if it does not drive responses. These efforts must be coupled with vigorous and targeted dissemination plans to convey the uniqueness of the local epidemic to key programmatic policy planners, communities and budget authorities in a clear and unambiguous manner. It is critical to counter the deeply ingrained generalized/concentrated paradigm at the highest levels, which so often misdirects prevention, fails to address key population needs, keeps key population resources constrained and gives stigma and discrimination additional breathing room. Regardless of region, the data and models demonstrate the urgent need to refocus local responses on the populations at the greatest risk if AIDS is to end by 2030.

## Acknowledgements

The authors gratefully acknowledge the hard work and support of the national AEM country teams in Bangladesh, Cambodia, Indonesia, Lao PDR, Malaysia, Myanmar, Nepal, Pakistan, the Philippines, Thailand and Viet Nam whose AEM-based national models for 2017 were essential inputs informing this article. Thanks are also due to Dr Swarup Sarkar and the World Health Organization South-East Asia Regional Office (WHO SEARO) who organized the think tank meeting on ‘Revisiting strategies for intervention among key populations’ in February 2018 that eventually led to this article.

### Financial support and sponsorship

The authors wish to thank WHO SEARO for their support of the recent scenario work for Asian countries used in this article. The authors further offer their deep gratitude to the Global Fund to Fight AIDS, Tuberculosis and Malaria; the Gates Foundation through Avenir Health; the Joint United Nations Programme on HIV/AIDS; Family Health International, the United States Agency for International Development and the World Bank who have supported the ongoing development and application of the AIDS Epidemic Model at national level over the last two decades. Their support made the national models used here possible.

### Conflicts of interest

There are no conflicts of interest.

## REFERENCES AND RECOMMENDED READING

Papers of particular interest, published within the annual period of review, have been highlighted as:

▪ of special interest
▪▪ of outstanding interest
